# Comparative Analysis of Mechanical Properties: Conventional vs. Additive Manufacturing for Stainless Steel 316L

**DOI:** 10.3390/ma17194808

**Published:** 2024-09-29

**Authors:** Constantin Alex Sumanariu, Cătălin Gheorghe Amza, Florin Baciu, Mihai Ion Vasile, Adrian Ionut Nicoara

**Affiliations:** 1 Department of Quality Engineering and Industrial Technologies, Faculty of Industrial Engineering and Robotics, National University of Science and Technology POLITEHNICA Bucharest, 060042 București, Romania; acata1@camis.pub.ro (C.G.A.); vasileionmihai@yahoo.com (M.I.V.); 2 Department of Strength Materials, Faculty of Industrial Engineering and Robotics, National University of Science and Technology POLITEHNICA Bucharest, 060042 București, Romania; florin.baciu@upb.ro; 3 Department of Science and Engineering of Oxide Materials and Nanomaterials, Faculty of Applied Chemistry and Materials Science, National University of Science and Technology POLITEHNICA Bucharest, 060042 București, Romania; adrian.nicoara@upb.ro

**Keywords:** tensile strength, SEM analysis, stainless steel 316L, additive manufacturing, conventional manufacturing, mechanical properties, microstructural analysis

## Abstract

This research investigates the tensile strength and microstructural properties of stainless steel 316L, comparing samples fabricated using additive manufacturing (AM) to those produced via conventional manufacturing techniques such as forging and casting using stainless steel 316L for its mechanical performance and corrosion resistance. Tensile tests revealed that AM samples had an ultimate tensile strength (UTS) of 650 MPa, a yield strength of 550 MPa and an elongation at break of 20%, and conventionally manufactured samples achieved a UTS of 580 MPa, a yield strength of 450 MPa and a higher elongation at break of 35%. The reduced ductility of AM samples is offset by their higher strength. Scanning electron microscopy (SEM) analysis showed that AM samples had a refined grain structure, with grain sizes ranging from 1 to 5 µm, whereas conventionally produced samples exhibited larger grain sizes of 10 to 20 µm, contributing to their increased ductility. This shows that while AM processes can give a rather high strength, the ductility property is simpler to attain with casting. Further work is needed to investigate post-processing techniques like hot isotropic pressing (HIP) and heat treatments for enhancing the ductility of AM parts as well as mechanical properties.

## 1. Introduction

Stainless steel 316L (SS316L) is a highly significant material in several industries such as aeronautical, medical devices and chemical processing, and it is so because it possesses exceptional corrosion resistance, mechanical qualities and biocompatibility. With the improvement of manufacturing technology, the conventional ways of making SS316L, such as casting and forging, are being progressively complemented or substituted by additive manufacturing (AM) methods like selective laser melting (SLM). AM has some identified benefits, like the capability to fabricate intricate shapes and minimise material inefficiency, which renders it an appealing choice for manufacturing high-performance parts. Nevertheless, the mechanical properties and microstructural characteristics of SS316L generated using AM might vary considerably compared to materials produced through conventional manufacturing methods. This discrepancy raises substantial concerns regarding the appropriateness of AM for crucial applications [1,2]. Presently, the study field exhibits an expanding collection of studies that concentrate on the comparison of the mechanical characteristics of SS316L produced by AM and conventional manufacturing methods. Several studies indicate that the materials resulting from AM have enhanced tensile strength because of their smaller grain structure [3,4]; however, other studies raise concerns regarding decreased ductility and the existence of residual stresses [5]. The contrasts in outcomes highlight the necessity of conducting a thorough assessment of both the tensile strength and microstructure to gain a complete understanding of the consequences associated with the utilisation of additive manufacturing techniques for SS316L components. The objective is to conduct a comparative analysis of the microstructure and tensile strength of SS316L samples that were produced using conventional and AM methods and understand the trade-offs between strength and ductility in these materials using tensile strength testing and SEM analysis. One of the potential findings is that SS316L could exhibit enhanced strength while experiencing decreased ductility.

Another good comparison could be from comparing UTS and yield strength from testing samples manufactured through AM and diffusion bonding, or other bonding techniques, balancing the trade-offs and identifying the optimal applications for each technique and material as highlighted by AlHazaa, A. et al. in their research on diffusion bonding techniques [6].

## 2. Materials and Methods

**Figure 1:**
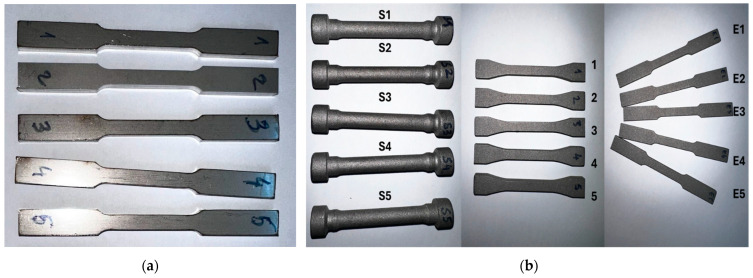
Stainless Steel 316L: (**a**) conventional manufacturing, (**b**) additive manufacturing.

Four sets of SS316L samples were prepared: The first one was made using conventional manufacturing methods (casting and forging) and the other using additive manufacturing (selective laser melting). The first set contains five samples, presented in Figure 1a; they were produced by melting the alloy and pouring it into sand moulds, followed by hot forging and annealing. Fifteen samples were fabricated using a commercial-grade SS316L powder presented in Figure 1b using SLM, the AM parts were acquired from a manufacturer named Sculpteo, Paris, France [7].

**Figure 2:**
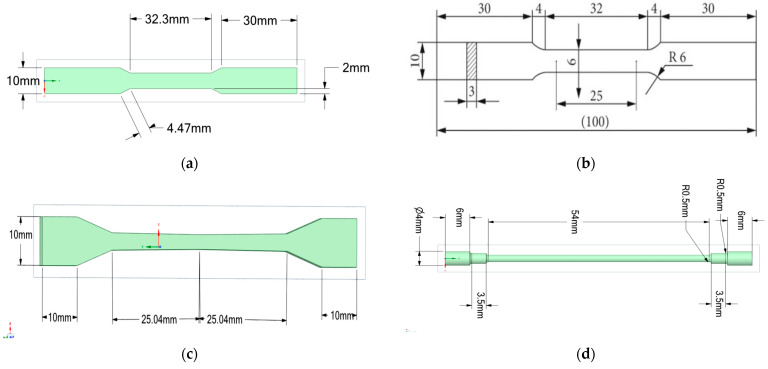
Sample dimensions for series. (**a**) E series sample dimensions; (**b**) conventional series sample dimensions; (**c**) numbered series sample dimensions; (**d**) S series sample dimensions.

The series samples are used in testing, as specified in Table 1, with E series samples measuring 100.9 × 3 × 10 mm, S series samples measuring 11 × 11 × 70 mm and numbered series samples measuring 75 × 4 × 10 mm as can be observed in Figure 2. The samples were manufactured using the laser melting technique used in additive manufacturing, and Stainless Steel 316L was selected for its corrosion resistance and mechanical properties [7].

The chemical composition for the samples and properties were taken from the manufacturer and are presented in Table 2 for both conventional manufactured samples and for AM samples.

Tensile testing was performed to assess the mechanical characteristics of both sets of samples. The experiment used a Forta Instron 8800/N as the equipment setup. The Instron machine is equipped with a very accurate load cell and fixtures that have the ability to securely hold the test specimens, ensuring reliable data collection.

The samples were labelled without any particular arrangement being used. The additive manufactured samples were evaluated in three series, each consisting of five samples. The conventional samples were examined in one series, also consisting of five samples.

The AM SS316L samples used for this study were produced using the EOS M280 machine. It utilises SLM technology which uses the systematic construction of components from metal powder through a layer-by-layer process and produces parts with microstructural details and specific mechanical properties, features that are important for the testing and analysis carried out in this research [7].

## 3. Results

The first AM set of samples, labelled S1 to S5, exhibits a modulus that varies between 0.01% and 0.1%, with corresponding values ranging from 155,889 MPa to 202,334 MPa. The modulus exhibits a coefficient of variance of 9.47%, indicating a moderate degree of variability in stiffness. The range of the yield stress is between 485 MPa and 644 MPa, with an average value of 565 MPa. The range of ultimate tensile strength is between 646 MPa and 852 MPa, with an average value of 761 MPa.

As specified in Table 3, the coefficient of variation for tensile strain was calculated at 1.16%. The fracture strain values range from 40.18% to 41.23%, with an average of 40.73%. The tensile strength at the point of fracture shows significant variance, with a coefficient of variation of 15.49%.

The second AM sample series, labelled from 1 to 5, exhibits somewhat reduced modulus values, yield stress values, ultimate tensile strength values and tensile strain values. The range of the ultimate tensile strength is between 738 and 755 MPa, with an average value of 653 MPa. The strain values are elevated, suggesting a high level of consistency, while the tensile strength at the point of fracture is greater, with an average value of 35.07%. The tensile strength at the point of fracture varies between 500 and 607 MPa, with an average of 438 MPa; this indicates a moderate level of variability in the way the material breaks. The results of the second series of tests are presented in Table 4.

The third AM sample Series, presented in Table 5, is labelled from E1 to E5. This series of tests presents a moderate level of rigidity compared to the first two series, with a modulus range of 150,600 MPa to 170,991 MPa and an average of 160,649 MPa. The yield stress varies between 427 MPa and 457 MPa, with an average of 446 MPa. The range of the ultimate tensile strength is between 603 MPa and 653 MPa, with an average of 629 MPa. The Poisson’s Ratio readings range from 0.312 to 0.339, with an average of 0.328, showing a moderate level of variation in the material’s capacity to bend laterally.

**Figure 3:**
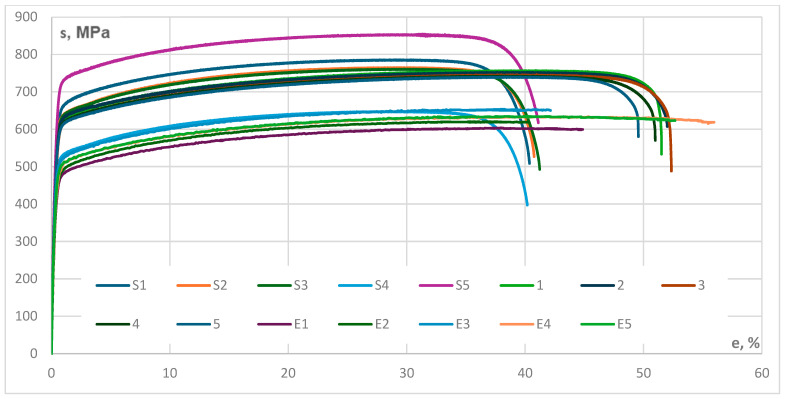
Stress–strain curves for AM Stainless Steel 316L samples.

The stress–strain curves that are presented in Figure 3 illustrate the mechanical response of stainless steel 316L samples produced using additive manufacturing when subjected to tensile stress for all three series of tests. The curves present data regarding mechanical characteristics including the elastic modulus, yield strength, ultimate tensile strength and ductility. The graph provides information on the material performance at the initial elastic region, yield point, plastic deformation, ultimate tensile strength, strain hardening, necking and fracture. By observing the variability in strength and ductility across the samples, a conclusion is that it is necessary to optimise AM process parameters to maintain material properties consistent. The results emphasise on how differences in the additive manufacturing process may affect the mechanical characteristics of 316L stainless steel.

The mechanical properties of 316L stainless steel samples that were manufactured using conventional methods are summarised in Table 6. The range of elastic modulus (E) values is between 130,527 MPa and 149,796 MPa, with an average value of 141,410 MPa. The Poisson’s ratio (Niu) varies between 0.302 and 0.357, with an average value of 0.328. The range of the ultimate tensile strength (UTS) is between 626.73 MPa and 677.04 MPa, with an average value of 657.25 MPa. The yield strength varies between 303.4 MPa and 350.36 MPa, with an average value of 332.68 MPa. The coefficient of variation suggests that the material’s reaction to applied force may be predicted. The samples demonstrate rigidity and durability, which are necessary for structural purposes. The yield strength indicates that the material will perform effectively in stress conditions commonly encountered in its applications. The Poisson’s ratio of stainless-steel indicates a uniform reaction to tensile pressures.

**Figure 4:**
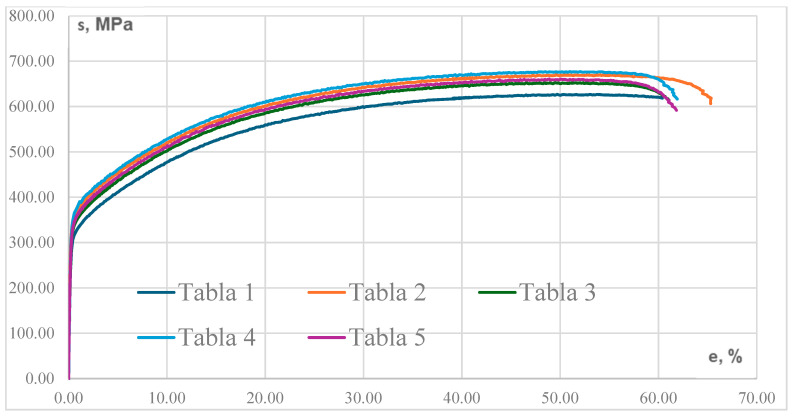
Stress–strain curves for conventional Stainless Steel 316L samples.

The mechanical properties of 316L stainless steel samples for the conventional T1–T5 series that were tested are represented by the stress–strain curve in Figure 4. The modulus slightly varies between samples which indicates stiffness consistency and shows sample stress and strain. The samples yielding stress also vary; therefore, each curve deviates from linearity at a slightly different stress level, moderately generating stability.

After yielding, plastic deformation occurs until the material reaches its maximum tensile strength. The samples reach higher UTS before necking and breaking. High material consistency.

Endpoint of curve shows material necking and fracture. The curves’ horizontal extension before dropping off shows constant ductility across samples.

After the tensile tests were performed, the resulting parts and a unfractured sample were analysed using a Scanning Electron Microscope (SEM) to observe and document identified microstructural changes and failure mechanisms induced by mechanical deformation. The analysis was performed at various magnifications to provide a understanding of the material’s behaviour under stress, how the grain structure is affected and fracture characteristics [8,9,10].

**Figure 5:**
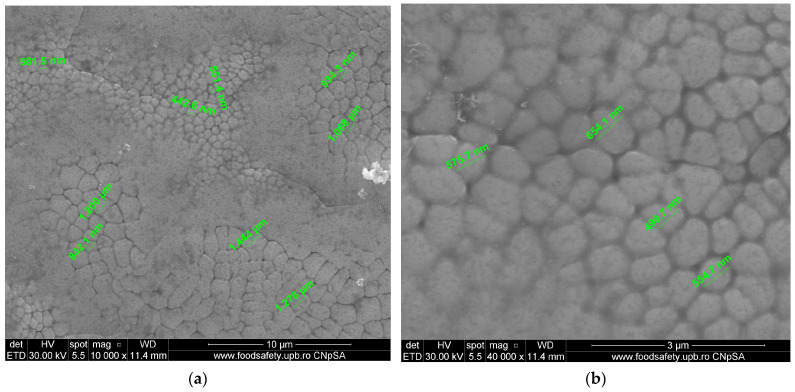
Unfractured AM sample—SEM analysis, (**a**) Unfractured AM sample 10,000× magnification, (**b**) Unfractured AM sample 40,000× magnification.

The scanning electron microscope (SEM) was utilised to examine the size and distribution of grains in an intact sample presented in Figure 5. The analysis has been conducted at magnifications of 10,000× and 40,000×. The SEM images at a magnification of 10,000× revealed a uniform grain structure; however, the diameters of the grains exhibited variation, suggesting diversity. The measured grain sizes that are documented in Table 7 indicate a non-uniform distribution, ranging from 442.6 nm to 1.444 µm. Under a magnification of 40,000×, scanning electron microscope (SEM) images revealed more detailed microstructural characteristics, including grain sizes ranging from 499.7 to 654.1 nanometres. At both magnifications, the grain size distribution was a mixture of fine and coarse grains.

**Figure 6:**
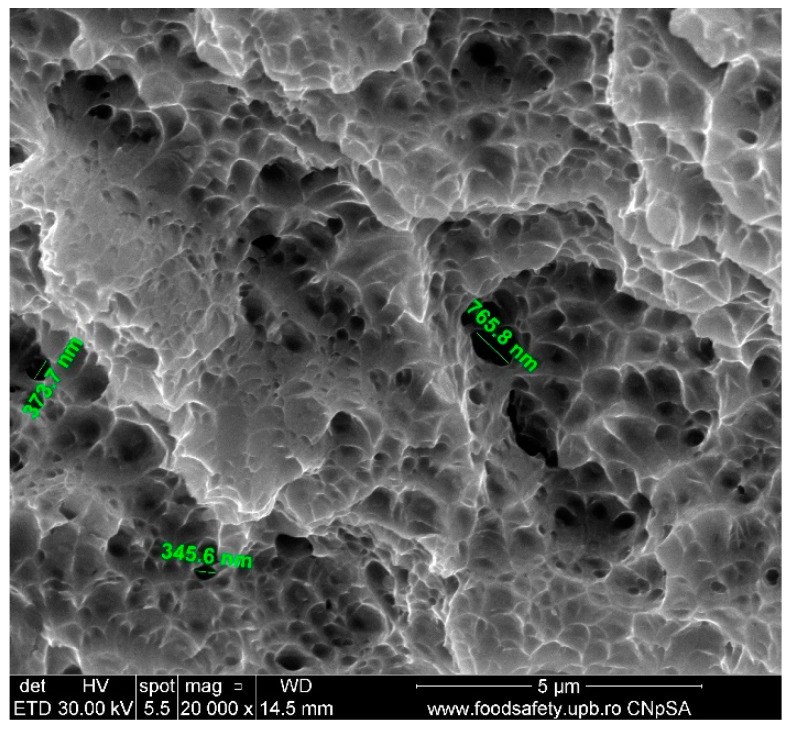
Fractured AM sample—SEM analysis of E5.

A scanning electron microscopy (SEM) investigation of the fragmented specimen E5 was conducted, and the results are centralised in Table 8. Sample E5, presented in Figure 6, demonstrates a ductile fracture mechanism that is characterised by the creation and merging of empty spaces. The scanning electron microscope (SEM) image, magnified at 20,000 times, reveals cavities of different diameters, suggesting substantial plastic deformation prior to failure. The existence of a coarse pitted fracture surface provides additional evidence of ductile fracture characteristics. The distribution of voids is not uniform, indicating the existence of stress concentrators that triggered the formation of voids. The empty spaces’ different dimensions show the presence of both minor and major deformation causes. The material’s microstructure, which includes factors such as grain size and the presence of inclusions or faults, can have an impact on the size and distribution of voids. The presence of voids and the appearance of the fracture surface suggest that the material possesses high fracture toughness and the capacity to absorb energy by undergoing plastic deformation.

**Figure 7:**
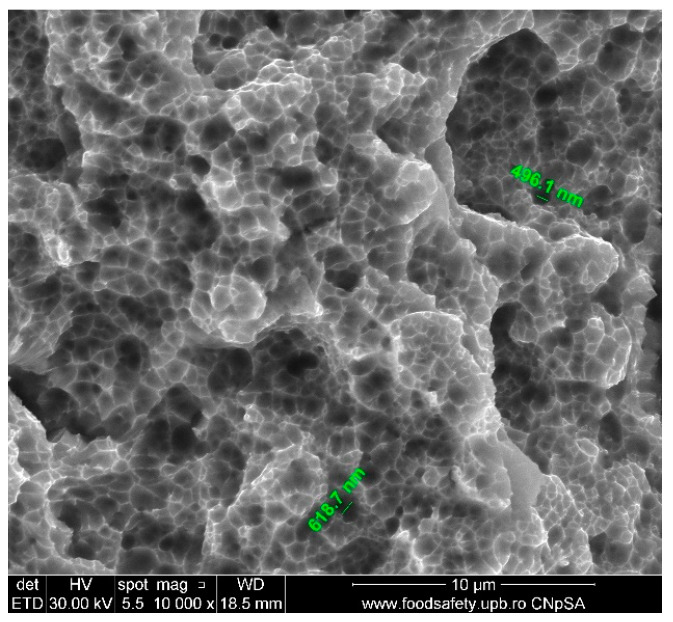
Fractured AM sample—SEM analysis of E1.

The fractured AM sample E1’s SEM image, presented in Figure 7 and described in Table 9, shows the existence of voids on the fracture surface. These voids are distinctive characteristics of ductile fracture. The diameters of the voids are 486.1 nm and 618.7 nm, indicating a precise microstructural response to the stress exerted. The fracture surface is coarse and indented, which is representative of a ductile fracture process. The presence of tiny voids indicates a high fracture toughness, which means that the material is resistant to the initiation and spread of cracks. The intricate microstructure, characteristic of additive manufacturing procedures, is responsible for the observed void dimensions, thereby improving the material’s mechanical characteristics. These findings are essential for comprehending the performance of AM stainless steel 316L in applications where mechanical integrity is of utmost importance.

**Figure 8:**
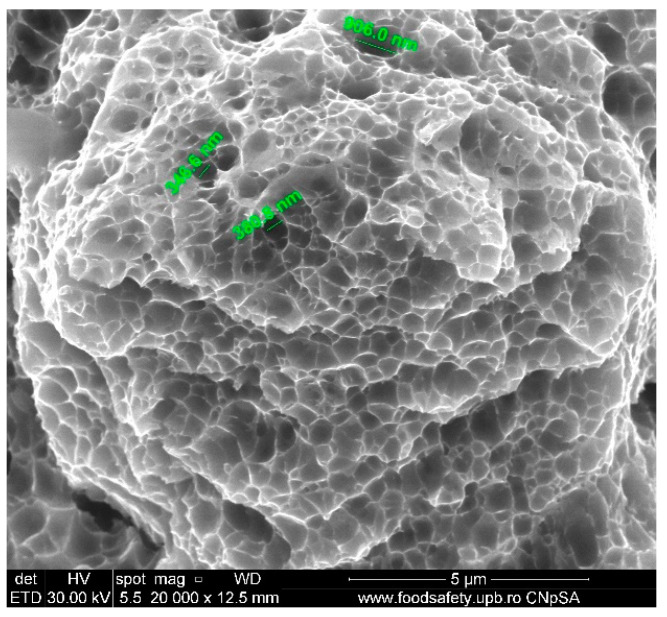
Fractured AM sample—SEM analysis of E2.

Figure 8 of the fractured AM Sample E2 demonstrates the presence of voids on the fracture surface. These voids are distinctive characteristics of ductile fracture. The diameters of the voids, as presented in Table 10, are 486.1 nm and 618.7 nm, indicating a precise microstructural response to the stress applied. The fracture surface is grainy and dented, which is consistent with the ductile fracture process. The presence of tiny voids indicates a high fracture toughness, which means that the material is resistant to the initiation and spread of cracks.

**Figure 9:**
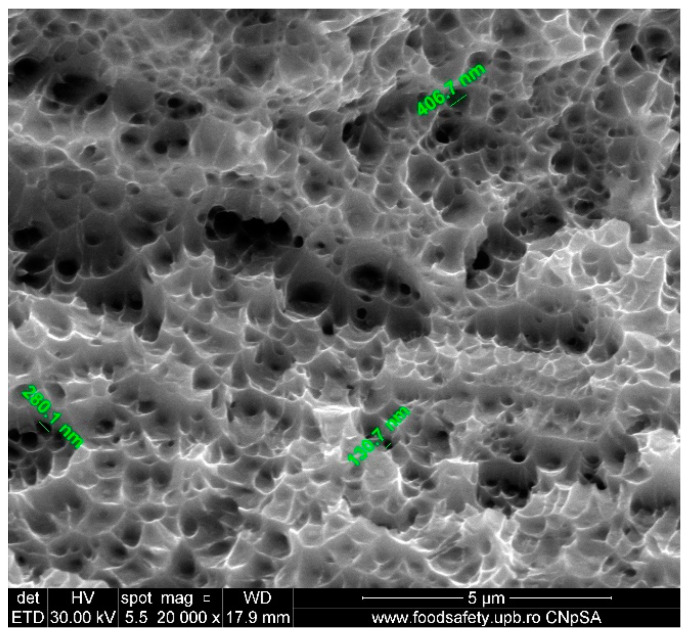
Fractured AM sample—SEM analysis of E3.

The void sizes observed in Figure 9 for Sample E3 varies between 130.7 nm and 406.7 nm, Table 11, indicating a small and refined microstructure compared to other fractured samples. The presence of minuscule cavities indicates that the material underwent localised plastic deformation on a smaller scale, which is characteristic of materials with greater strength and microstructures composed of fine grains; overall, this sample material provided better response to stress compared to previous samples.

The presence of small voids, especially the 130.7 nm void, suggests that the fracture in Sample E3 most likely started and spread through a ductile mechanism. The voids formed at a variety of small locations within the material. The presence of varying void sizes suggests that the material may have encountered different degrees of stress concentration along the fracture surface.

Voids of varying sizes, ranging from 130.7 nm to 406.7 nm, demonstrate the material’s ductility. These voids indicate the material’s capacity to undergo plastic deformation prior to fracturing, which is a feature of ductile materials. The discovered voids likely resulted from the merging of micro voids during the material’s deformation, ultimately causing it to fracture.

**Figure 10:**
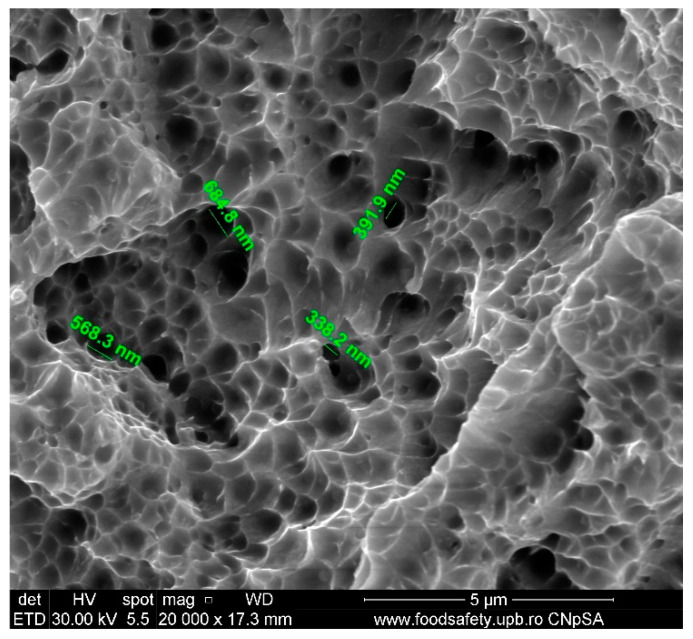
Fractured AM sample—SEM analysis of E4.

As can be observed in Figure 10 and documented in Table 12 for AM sample E4, the voids vary in size from 338.2 nm to 684.8 nm, suggesting a significant variation in the distribution of stress and strain on the fracture surface exhibiting clear characteristics of ductility.

**Figure 11:**
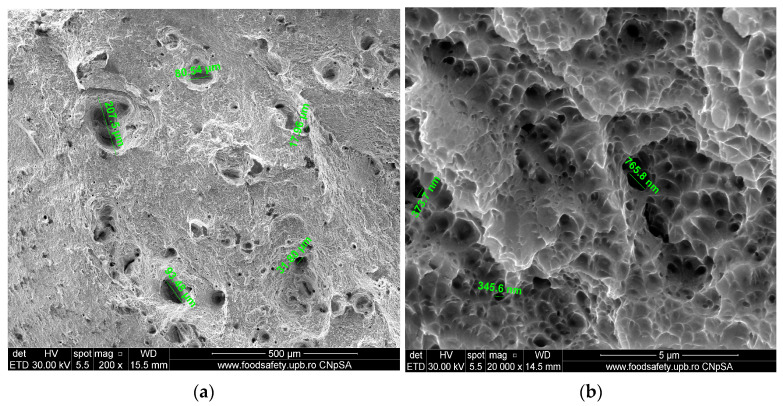
Fractured AM sample—SEM analysis of E5. (**a**) 200× magnification; (**b**) 20,000× magnification.

The broken AM Sample E5, presented in Table 13 also presents a fracture process with voids at various levels, indicating substantial distortion and stress accumulation. These findings, from Figure 11, align with previous structural findings on other fractured AM samples.

## 4. Discussion

**Figure 12:**
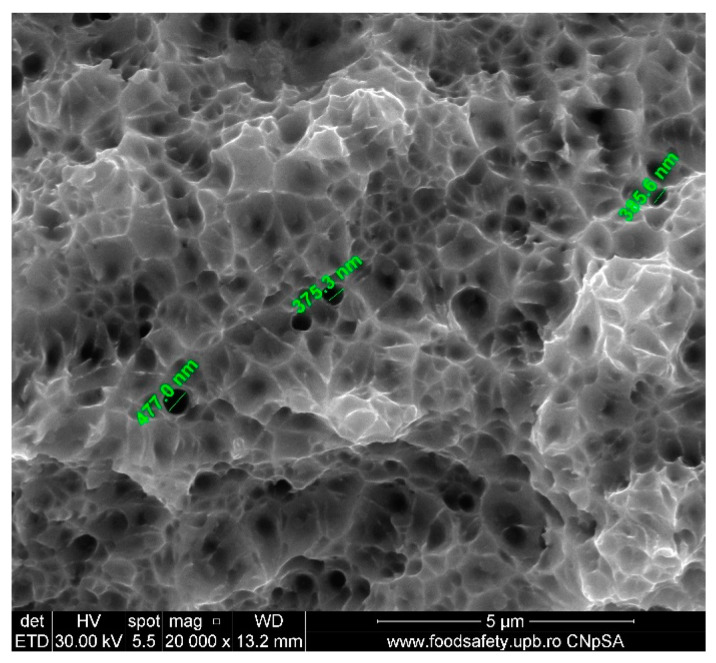
Fractured AM sample—SEM analysis of S2.

**Figure 13:**
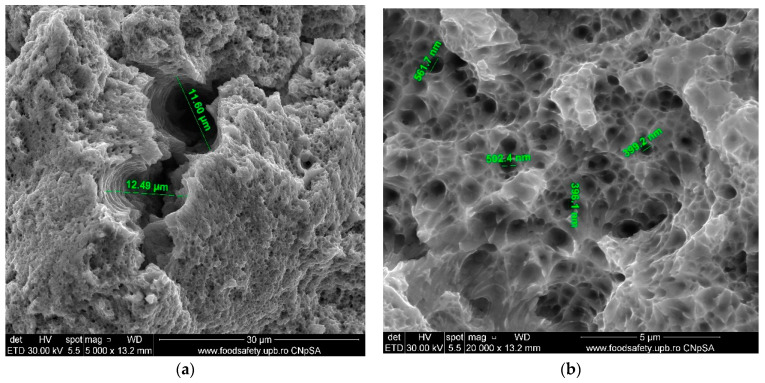
Fractured AM sample—SEM analysis of S3. (**a**) 5000× magnification; (**b**) 20,000× magnification.

**Figure 14:**
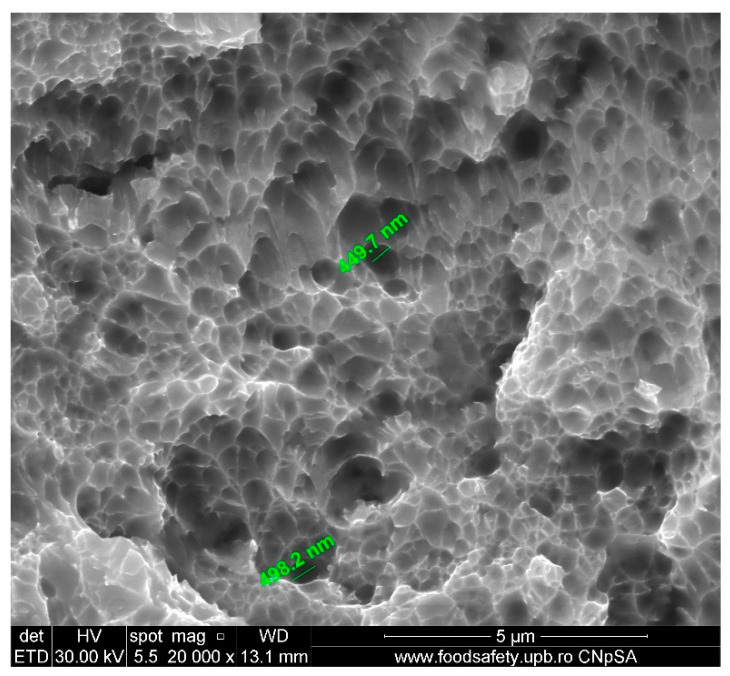
Fractured AM sample—SEM analysis of S4, 20,000× magnification.

**Figure 15:**
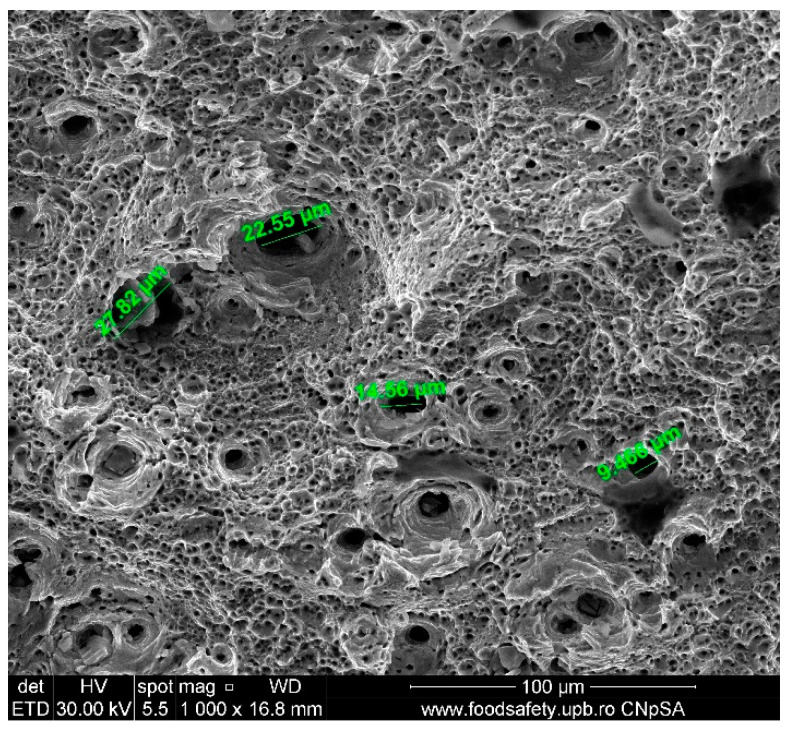
Fractured AM sample—SEM analysis of Sample 1, 1000× magnification.

**Figure 16:**
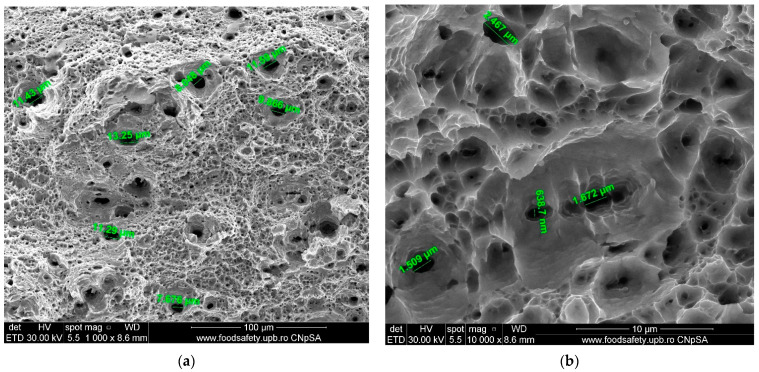
Fractured AM sample—SEM analysis of Sample 2. (**a**) 1000× magnification; (**b**) 10,000× magnification.

**Figure 17:**
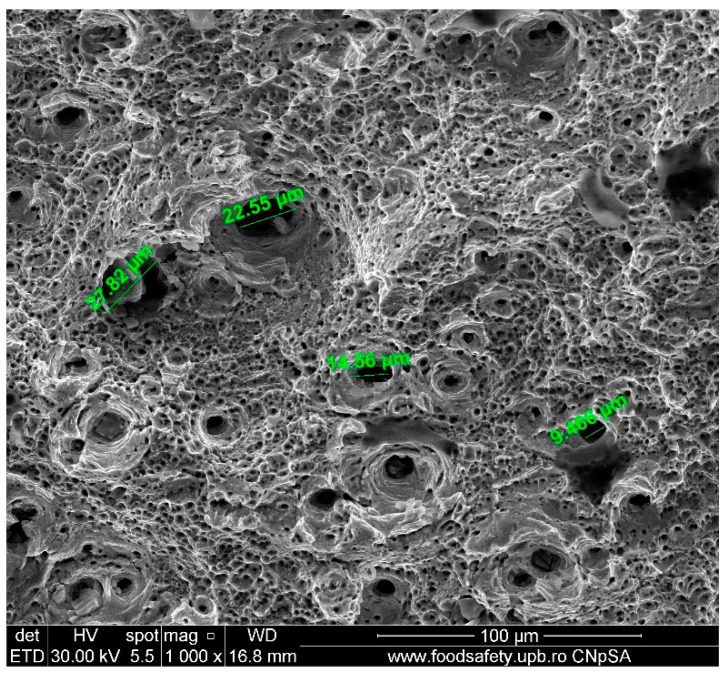
Fractured AM sample—SEM analysis of Sample 3, 500× magnification.

**Figure 18:**
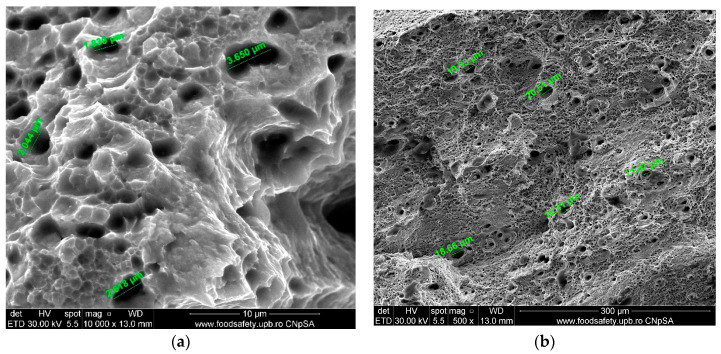
Fractured AM sample—SEM analysis of Sample 4. (**a**) 500× magnification; (**b**) 10,000× magnification.

**Figure 19:**
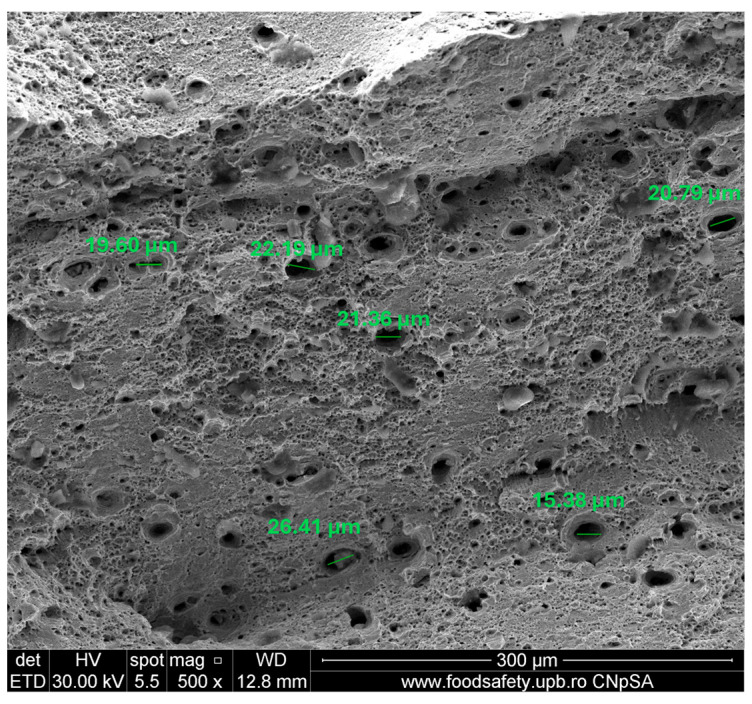
Fractured AM sample—SEM analysis of Sample 5, 500× magnification.

The stress–strain curves and SEM images, presented in Figure 5, Figure 6, Figure 7, Figure 8, Figure 9, Figure 10, Figure 11, Figure 12, Figure 13, Figure 14, Figure 15, Figure 16, Figure 17, Figure 18 and Figure 19, of the fractured samples that were produced using AM exhibit superior tensile strength and yield strength compared to conventionally manufactured samples. The SEM pictures present a more refined grain structure, suggesting the typical characteristics of AM processes showcasing a higher strength and they also exhibited reduced ductility, as indicated by lower values of elongation at break. While the additive manufacturing technique enhances strength, it was observed that based on the grain structure distribution it may lead to residual stresses or faults that compromise ductility. As documented in Table 7, Table 8, Table 9, Table 10, Table 11, Table 12, Table 13, Table 14, Table 15, Table 16, Table 17, Table 18, Table 19, Table 20 and Table 21, the presence of empty spaces, voids and irregularities in the microstructure contributed to the material’s reduced flexibility prior to failure as was confirmed by SEM analysis of fractured AM samples. Samples created through conventional manufacturing techniques showed higher flexibility despite having a lower level of tensile strength.

ANOVA, short for Analysis of Variance, is a statistical technique used to assess if there exists a documentable disparity among the means of three or more groups [11,12].

The data were gathered, segregated into groups and calculated using the F-statistic and *p*-value. A *p*-value below 0.05 indicates significant differences in tensile properties across the groups, indicating that these differences were not random but due to the specific conditions of the samples [13].

**Figure 20:**
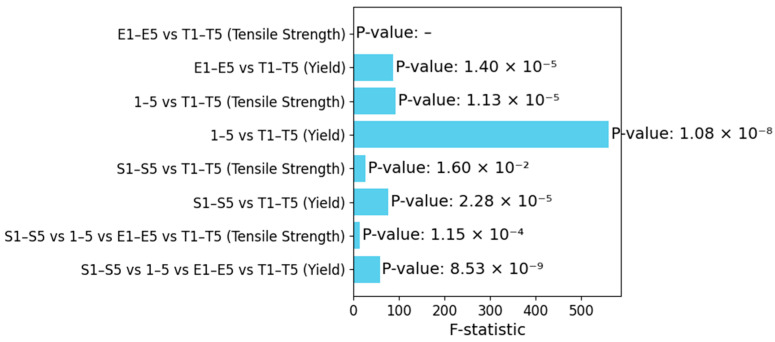
F-Statistic Comparison Across Different Sample Groups.

The analysis of variance which is presented in Table 22 and illustrated in Figure 20, was performed for the results of the tensile stress tests of the samples. The analysis compared the tensile stress at the point of yield and the tensile stress at the point of maximum strength in the previously presented sample series. Differences in the yield strengths between all the sample comparisons were identified, and the F-statistic was highest between the 1–5 AM series and the T1–T5 conventional series. The tensile stress at the point of tensile strength has variations, with *p*-values often above those for yield stress [13,14].

## 5. Conclusions

The comparison of SS316L samples produced using conventional and additive tech-niques documented the compromises associated with the manufacturing technologies. By creating a more refined microstructure, additive manufacturing improves materials’ ten-sile strength and yield strength coming at the expense of reduced ductility. Conventional production techniques produce materials with lower strength but higher ductility; this means that it is important when choosing the appropriate production method to adapt the manufacturing techniques to the individual requirements of the application. The use of AM offers benefits in terms of high strength for specific uses. However, for applications that require a balance of strength and flexibility, traditional production methods could prove more suitable. Future research is required to focus on improving post-processing techniques for additive manufacturing samples to mitigate the negative effects of reduced ductility.

The relatively fast cooling and solidification rates in AM provide finer grain structure and greater dislocation density, therefore affecting the tensile strength.

Conventionally produced SS316L shows a more evenly distributed grain structure with larger grain sizes compared to the microstructure of AM SS316L which shows finer grains and columnar grain growth aligned in the build direction due to rapid cooling rates of the AM process. While grain refinement in AM samples results in higher strength but decreased ductility, the AM samples have greater porosity and anisotropy than conventionally produced components, which influences mechanical parameters including tensile strength and ductility.

Using the SLM AM process, the fast cooling and solidification speeds inherent in the process define the refined grain architecture improving the microstructure compared to conventional methods. Also, the layer-by-layer construction that creates steep thermal gradients, and promotes columnar grain growth in the build direction also improves the material properties.

The presence of microstructural defects like porosity, anisotropy, and residual stresses lead to a reduced ability for plastic deformation, making the material more brittle. While fine grain structures from rapid solidification increase strength, they also limit the material’s capacity for elongation before failure, resulting in lower ductility.

Larger grain sizes in conventionally manufactured stainless steel 316L are correlated with improved ductility because they allow for greater dislocation movement and plastic deformation. This results in the material being able to stretch and absorb more strain before fracturing, as larger grains reduce grain boundary strengthening, which can contribute to the higher ductility observed [15,16].

When considering the results and the conclusions specific limitations must be mentioned:

- The results are for this specific manufacturing process (SLM), for this particular material (SS316L) with the material composition mentioned in this article.
- The degree of porosity for each sample may vary based on type and model of machine and materials used in the manufacturing process which may influence the values [16].
- The study did not focus particularly on post-processing techniques, only shot peening was used for the post-processing of the samples presented in this study.

As AM technology evolves, and post-processing techniques improve there is a high chance of using AM parts and/or materials used more and more in the nuclear sector for more important components. This would facilitate on-site fabrication of specific components and on-site qualification for the specific environment to be used.

D’Andrea noticed similar behaviour of AM SS316L material properties and documented in their paper that depending on post-processing procedures, the treatments may have a significant impact on hardness of the material and that the austenite stability and build direction affect the fatigue properties of the specimens [16].

Kedizora et al. also documented that the SS316L material manufactured through the EOS machine models via SLM (DMLS—Direct Metal Laser Sintering) process models presented the greatest fatigue strength when comparing two specimens and identifies that the heat-treated material does not necessarily present increased ductility [17]. Jeyaprakash focused on the process parameters and how they influenced the microstructural orientation of SS316L during the melt pool formation [18]. These findings need to be explored more to understand what specific post-processing treatment is best for increasing ductility with minimal compromise of other properties.

Future work should focus on identifying the optimum balance between energy density, laser power, speed, layer thickness and cooling rates. Phase analysis and microstructural characterisation should be used to validate the optimal selection of process parameters [19,20].

Another topic to be explored would be to fabricate radial bimetallic structures that present distinctive opportunities for designing and manufacturing parts with superior mechanical properties as described by Dash A. et al. in their paper [21].

Identified post-processing strategies also comprise of hot isostatic pressing (HIP), which lowers internal porosity and increases material density and heat treatment help with the release of residual stresses. Shot peening or laser remelting also represent methods that can help to reduce stress concentrations and enhance surface quality.

The specific application requirements that were identified so far that might influence the choice between AM and conventional manufacturing techniques for SS316L are:

- Geometry requirements;
- Material properties requirements;
- Cost of maintenance and production;
- Possibility of manufacturing unique parts and prototypes.

The choice between AM and conventional manufacturing for SS316L depends on several considerations, particularly in specialised applications like medical implants, aerospace or nuclear technologies.

Overall SS316L represents a decent material and AM a suitable manufacturing method to be used in the industrial environment more specifically in the nuclear sector. The differences in sample results are representative for the variation in sizes and shapes of the samples and show considerable improvement compared to conventional samples. Further research is required to provide a standardised test plan for such materials and methods to be used in the nuclear field.

## Figures and Tables

**Table 1:** Specifications of AM SS316L samples.

Specification	Process	Material	Finish	Plan	Size
E series sample	Laser Melting (Metal)	Stainless Steel 316L	Shot peened	Standard plan	100.9 × 3 × 10 mm
S series sample	Laser Melting (Metal)	Stainless Steel 316L	Shot peened	Standard plan	11 × 11 × 70 mm
Numbered series samples	Laser Melting (Metal)	Stainless Steel 316L	Shot peened	Standard plan	75 × 4 × 10 mm

**Table 2:** AM and conventional SS316L comparative table [7].

Property	316L Stainless Steel AM Properties	ASM SS316L Properties (Conventional)
Designation Chemical Composition (Weight%)	EU: 1.4404	EU: 1.4404
	UNS: S31603	UNS: S31603
	Fe: Balance	Fe: Balance
	Cr: 16–18	Cr: 16–18
	Ni: 10–14	Ni: 10–14
	Mo: 2–3	Mo: 2–3
	Mn: <2	Mn: <2
	N: <0.1	N: <0.1
	Si: <0.75	Si: <0.75
	P: <0.045	P: <0.045
	C: <0.03	C: <0.03
	S: <0.03	S: <0.03
Mechanical Properties	Ultimate Tensile Strength (UTS): 650 ± 50 MPa	Ultimate Tensile Strength (UTS): 485 MPa (minimum)
	Yield Strength (Rp 0.2%): 550 ± 50 MPa	Yield Strength (Rp 0.2%): 170 MPa (minimum)
	Elongation at Break: 45%	Elongation at Break: 40% (minimum)
	Young’s Modulus: 170 GPa	Young’s Modulus: 193 GPa
	Notched Charpy Impact Strength: 30 J/cm^2^	Notched Charpy Impact Strength: Typically, not specified
Thermal Properties	Thermal Conductivity (at 20 °C): 16.2 W/m·K	Thermal Conductivity (at 20 °C): 16.3 W/m·K
	Specific Heat Capacity (at 20 °C): 500 J/kg·K	Specific Heat Capacity (at 20 °C): 500 J/kg·K
	Melting Point: 1380 °C	Melting Point: 1375–1400 °C

**Table 3:** S1–S5 series sample results.

Sample ID	Modulus (Segment 0.01–0.1%)	Tensile Stress at Yield (Offset 0.2%)	Tensile Stress at Tensile Strength	Tensile Strain at Tensile Strength	Tensile Strain at Break (Standard)	Tensile Stress at Break (Standard)
	(MPa)	(MPa)	(MPa)	(%)	(%)	(MPa)
S1	186,319.333	574.7482	784.4247	28.93	40.3689	509.0764
S2	193,649.867	569.6427	763.8884	29.136	40.75689	526.9195
S3	187,343.527	553.1412	759.7135	29.471	41.23047	492.989
S4	155,889.32	485.9432	646.7994	28.624	40.1875	397.0341
S5	202,334.445	644.0778	852.0469	29.351	41.10901	617.4066
Mean	185,107.298	565.5106	761.3746	29.102	40.73055	508.6851
Coefficient of Variation	9.47290412	9.987128	9.71601	1.1615	1.111309	15.49951

**Table 4:** 1–5 series sample results.

Sample ID	Modulus (Segment 0.01–0.1%)	Tensile stress at Yield (Offset 0.2%)	Tensile Stress at Tensile Strength	Tensile Strain at Tensile Strength	Tensile Strain at Break (Standard)	Tensile Stress at Break (Standard)
	(MPa)	(MPa)	(MPa)	(%)	(%)	(MPa)
1	177,455.141	545.8348	755.6332	39.46	51.52101	578.1633
2	178,026.049	556.5818	750.9918	38.821	52.00457	607.2736
3	175,913.402	535.8724	743.8113	38.596	52.35189	500.8993
4	185,568.796	545.8407	741.5116	38.29	50.98168	588.1709
5	174,619.689	549.6757	738.7468	38.431	49.55344	606.7018
Mean	158,664.752	486.1501	653.9948	25.111	35.07066	438.23
Coefficient of Variation	2.39349702	1.372351	0.936161	1.185	2.136545	7.620517

**Table 5:** E1–E5 series sample results.

Sample ID	Modulus (Segment 0.01–0.1%)	Tensile Stress at Yield (Offset 0.2%)	Tensile Stress at Tensile Strength	Poisson’s Ratio (ν)
E1	150,600.528	427.14	603.547	0.335
E2	164,079.901	433.35	620.234	0.335
E3	170,991.442	474.8	653.508	0.319
E4	159,019.197	438.1	633.528	0.339
E5	158,556.243	457.12	634.857	0.312
Mean	160,649.462	446.102	629.1348	0.3281
Coefficient of Variation	4.68661706	4.385793	2.952363	3.5769

**Table 6:** T1–T5 series sample results.

Sample ID	Modulus (Segment 0.01–0.1%)	Tensile Stress at Yield (Offset 0.2%)	Tensile Stress at Tensile Strength	Poisson’s Ratio (ν)
T1	145,121.962	303.4	626.7325885	0.307146911
T2	148,756.53	350.36	669.66886	0.3238474
T3	130,527.28	328.6	652.33477	0.3482013
T4	149,795.5	347.71	677.0448	0.302288
T5	132,848.7	333.32	660.4563	0.357127
Mean	141,409.999	332.678	657.247	0.328
Coefficient of Variation	6.421155	5.649306	2.957945	7.426299

**Table 7:** Unfractured AM sample grain size.

Observed Grain Size for Unfractured Sample	Magnification
581.5 nm	10,000×
442.6 nm	10,000×
551.4 nm	10,000×
1.200 µm	10,000×
842.1 nm	10,000×
1.444 µm	10,000×
1.278 µm	10,000×
1.068 µm	10,000×
934.3 nm	10,000×
575.7 nm	40,000×
654.1 nm	40,000×
499.7 nm	40,000×
554.7 nm	40,000×

**Table 8:** Fractured AM sample E5 void dimension at 20,000× magnification.

Observed Void Dimensions for Fractured Sample	Magnification
373.7 nm	20,000×
345.6 nm	20,000×
765.8 nm	20,000×
296.7 nm	20,000×
412.7 nm	20,000×

**Table 9:** Fractured AM sample E1 void dimension.

Observed Void Dimensions for Fractured Sample	Magnification
486.1 nm	10,000×
618.7 nm	10,000×

**Table 10:** Fractured AM sample E2 void dimension.

Observed Void Dimensions for Fractured Sample	Magnification
906.0 nm	20,000×
346.6 nm	20,000×
380.8 nm	20,000×

**Table 11:** Fractured AM sample E3 void dimension.

Observed Void Dimensions for Fractured Sample	Magnification
280.1 nm	280.1 nm
130.7 nm	130.7 nm
406.7 nm	406.7 nm

**Table 12:** Fractured AM sample E4 void dimension.

Observed Void Dimensions for Fractured Sample	Magnification
391.9 nm	20,000×
338.2 nm	20,000×
684.8 nm	20,000×
568.3 nm	20,000×

**Table 13:** Fractured AM sample E5 void dimension at 200× magnification.

Observed Void Dimensions for Fractured Sample	Magnification
80.54 µm	200×
17.96 µm	200×
31.80 µm	200×
93.48 µm	200×
207.5 µm	200×

**Table 14:** Fractured AM sample S2 void dimension.

Observed Void Dimensions for Fractured Sample	Magnification
477.0 nm	20,000×
375.3 nm	20,000×
385.6 nm	20,000×

**Table 15:** Fractured AM sample S3 void dimension.

Observed Void Dimensions for Fractured Sample	Magnification
11.60 µm	5000×
12.49 µm	5000×
502.4 nm	20,000×
561.7 nm	20,000×
396.1 nm	20,000×
399.2 nm	20,000×
11.60 µm	5000×

**Table 16:** Fractured AM sample S4 void dimension.

Observed Void Dimensions for Fractured Sample	Magnification
449.7 nm	20,000×
498.2 nm	20,000×

**Table 17:** Fractured AM sample 1 void dimension.

Observed Void Dimensions for Fractured Sample	Magnification
22.55 µm	1000×
27.82 µm	1000×
14.56 µm	1000×
9.466 µm	1000×

**Table 18:** Fractured AM sample 2 void dimension.

Observed Void Dimensions for Fractured Sample	Magnification
22.55 µm	1000×
11.43 µm	1000×
13.25 µm	1000×
11.29 µm	1000×
7.676 µm	1000×
8.048 µm	1000×
11.59 µm	1000×
9.866 µm	1000×
1.509 µm	10,000×
638.7 µm	10,000×
1.672 µm	10,000×
2.467 µm	10,000×

**Table 19:** Fractured AM sample 3 void dimension.

Observed Void Dimensions for Fractured Sample	Magnification
17.94 µm	500×
23.24 µm	500×
48.71 µm	500×
12.60 µm	500×

**Table 20:** Fractured AM sample 4 void dimension.

Observed Void Dimensions for Fractured Sample	Magnification
19.93 µm	500×
20.51 µm	500×
18.66 µm	500×
16.41 µm	500×
11.87 µm	500×
2.044 µm	10,000×
2.618 µm	10,000×
1.839 µm	10,000×
3.650 µm	10,000×

**Table 21:** Fractured AM sample 5 void dimension.

Observed Void Dimensions for Fractured Sample	Magnification
19.60 µm	500×
22.19 µm	500×
21.36 µm	500×
26.41 µm	500×
15.38 µm	500×

**Table 22:** ANOVA sample analysis.

Comparison	Property	F-Statistic	*p*-Value
S1–S5 vs. 1–5 vs. E1–E5 vs. T1–T5	Tensile Stress at Yield	57.61	8.53 × 10^−9^
S1–S5 vs. 1–5 vs. E1–E5 vs. T1–T5	Tensile Stress at Tensile Strength	13.60	1.15 × 10^−4^
S1–S5 vs. T1–T5	Tensile Stress at Yield	76.50	2.28 × 10^−5^
S1–S5 vs. T1–T5	Tensile Stress at Tensile Strength	9.27	0.016
1–5 vs. T1–T5	Tensile Stress at Yield	559.58	1.08 × 10^−8^
1–5 vs. T1–T5	Tensile Stress at Tensile Strength	92.58	1.13 × 10^−5^
E1–E5 vs. T1–T5	Tensile Stress at Yield	87.40	1.40 × 10^−5^
E1–E5 vs. T1–T5	Tensile Stress at Tensile Strength	-	-

## Data Availability

The data that support the findings of this study are available from the corresponding author upon reasonable request. The data are not publicly available due to privacy or ethical restrictions.
